# Regulation of the survival and differentiation of hepatic stem/progenitor cells by acyclic retinoid

**DOI:** 10.1186/s13287-015-0099-9

**Published:** 2015-05-29

**Authors:** Akihide Kamiya

**Affiliations:** Department of Molecular Life Sciences, Tokai University School of Medicine, 143 Shimokasuya, Isehara, Kanagawa 259-1193 Japan

## Abstract

During embryonic liver development, hepatic stem/progenitor cells (HpSCs) have a high proliferative ability and bipotency to differentiate into hepatocytes and cholangiocytes. Retinoic acid is a derivative of vitamin A and is involved in the proliferation and differentiation of stem/progenitor cells in several tissues. However, whether retinoic acid regulates the characteristics of HpSCs in the normal liver is still unknown. A recent study has shown that acyclic retinoid regulates the survival and proliferation of HpSCs derived from mouse foetal liver. Acyclic retinoid suppressed the expansion of CD29^+^CD49f^+^ HpSCs through the induction of hepatocytic differentiation and progression of apoptosis.

## Introduction

The liver is the largest organ in the adult human body and is important for maintaining homeostasis. Adult hepatocytes and liver parenchymal cells express genes and enzymes for several metabolic functions. In addition, the liver includes several non-parenchymal cells such as cholangiocytes, sinusoidal endothelial cells, stellate cells, and Kupffer cells. Hepatocytes and cholangiocytes are differentiated from the same source: the stem/progenitor cells in embryonic livers. Guan and colleagues, in a recent article in *Stem Cell Research & Therapy*, investigated a new mechanism regulating the survival and differentiation of these stem/progenitor cells by retinoic acid signals [1].

There are two types of liver progenitor cells: foetal hepatic progenitor cells and postnatal/adult hepatic progenitor cells. Liver formation begins with the specification of the foetal hepatic progenitor cells derived from the foregut endoderm. During mid- to late-foetal liver development, hepatic progenitor cells differentiate into hepatocytes and cholangiocytes. It has been reported that the interaction between hepatic cells and hematopoietic cells is important for foetal liver maturation [2]. Moreover, in cases of serious injury to the adult liver, such as that induced by retrorsine or 2-acetylaminofluorine treatment in combination with partial hepatectomy, the number of characteristic progenitor-like cells expressing both cholangiocellular and hepatocellular markers increases in periportal regions [3].

These foetal and adult hepatic progenitor cells are well characterized by specific cell surface antibodies and cell purification methods such as flow cytometry. Suzuki and colleagues [4] found CD29 and CD49f to be specific markers and established *in vitro* colony formation assays of hepatic progenitor cells. In addition, markers such as Dlk, Liv2, E-cadherin, CD13, and CD133 are reported to be specific markers of foetal hepatic progenitor cells. CD13 and CD133 are also expressed in progenitor cells derived from adult livers. Recent studies revealed that long-term proliferative hepatic progenitor cells exist in mouse and human adult livers. LGR5^+^ and EpCAM^+^ progenitor cells can form cystic structures and expand *in vitro* for several months [5, 6]. An *in vitro* culture system of purified cells using antibodies against these cell surface antigens is useful for analyses of molecular mechanisms regulating the proliferation and differentiation of hepatic progenitor cells.

It is known that, in addition to stem/progenitor cells in the normal liver, cancer stem-like cells are present in hepatocellular carcinomas (HCCs) or other types of cancers. Cancer stem-like cells have characteristics similar to those exhibited by somatic stem cells and are important for tumour initiation, metastasis, and recurrence. Retinoic acid is a natural derivative of vitamin A and regulates its target genes by binding to nuclear receptors such as retinoic acid receptors (RARs) and retinoid X receptors (RXRs). Retinoic acid is important for the differentiation and proliferation of stem/progenitor cells in both somatic and cancer tissues. For example, retinoic acid is used for the differentiation of pluripotent stem cells into mature functional cells. In addition, acyclic retinoid (ACR) exhibited anti-HCC effects in several hepatoma models [7, 8]. In contrast, liver regeneration *in vivo* is induced by the addition of all-trans retinoic acid (ATRA) [9]. Thus, the exact effects of retinoic acid signals on normal hepatic stem/progenitor cells (HpSCs) in somatic tissues remain unknown.

Guan and colleagues [1] stated that the addition of ACR regulated the survival and differentiation of HpSCs derived from mouse embryonic liver. Peretinoin is a synthetic ACR that has been known to reduce the risk of HCC recurrence or death. In addition, peretinoin inhibits the replication and release of hepatitis C virus [10]. Guan and colleagues isolated HpSCs by using specific cell surface antigens (CD29 and CD49f) and flow cytometry and cultured them in a low-density colony assay system. These cells have a high colony-forming ability and express several retinoic acid-binding receptors - namely, Rarα, Rxrα, and Rxrβ - at higher levels than those expressed by adult hepatocytes. The addition of peretinoin significantly reduced the total number of cells and colony formation by HpSCs in a dose-dependent manner. After peretinoin treatment, the clonal expansion of HpSCs was suppressed and the colony size was significantly reduced. In addition, the expression of the cell cycle gene cyclin D1 was decreased whereas the expression of cdk inhibitor p21^Cip1^ was upregulated by the addition of peretinoin. These results suggested that ACR inhibits the expansion of stem/progenitor cells derived from embryonic livers. This cell cycle arrest is involved in the differentiation of HpSCs. The authors found that ACR induced maturation of HpSCs into hepatocytic cells. The addition of peretinoin induced the expression of cells positive for the hepatocyte marker albumin in this culture system. In contrast, the HpSC marker genes such as α-feto protein, Cd44, and Dlk were significantly downregulated by peretinoin. The expression of cytokeratin 19, a cholangiocyte marker, was also decreased, suggesting that the addition of ACR promoted hepatic differentiation, but not cholangiocytic differentiation, of HpSCs. In addition, ACR regulated HpSC survival via apoptosis.

Guan and colleagues have provided evidence that retinoic acids regulate proliferation and differentiation of HpSCs during liver development. Whether retinoic acids ATRA and ACR positively or negatively affect cell proliferation in normal liver and hepatic carcinoma tissues is controversial. The present study clearly showed that ACR suppressed the proliferation and survival of stem/progenitor cells in normal embryonic liver tissues. A synthetic retinoid ACR might have a cell signal cascade that is different from that of natural retinoic acids. Hepatic cancer stem cells also exhibit stem/progenitor characteristics similar to those exhibited by normal HpSCs. Therefore, this study suggested that ACR not only plays the role of an anti-tumour drug in HCC but also inhibits tumour-initiating cells such as hepatic cancer stem cells.

## Abbreviations

ACR: Acyclic retinoid
ATRA: All-trans retinoic acid
HCC: Hepatocellular carcinoma
HpSC: Hepatic stem/progenitor cell
RAR: Retinoic acid receptor
RXR: Retinoid X receptor
